# Contact Tracing and Prevention of Secondary Measles Transmission in a Pediatric Acute Care Hospital — Los Angeles County, July 2024

**DOI:** 10.1017/ash.2025.203

**Published:** 2025-09-24

**Authors:** Jordan Braunfeld, Edahrline Salas, Lingao Oliver-Donn, Kathy Trigueiro, Karyn Wong, Michael Smit, Susan Wu, Christine Benjamin, Sherry Yin, Zachary Rubin, Andrea Kim, Nava Yeganeh

**Affiliations:** 1 Centers for Disease Control and Prevention; 2 Children’s Hospital Los Angeles; 3 Children’s Hospital Los Angeles/USC Keck School of Medicine; 4 LA County Department of Public Health; 5 Los Angeles County Department of Public Health; 6 David Geffen School of Medicine at UCLA

## Abstract

**Background:** Measles cases have been increasing in the United States and globally. However, the nonspecific presentation and ability to mimic and coexist with other common infections can delay diagnosis. During July 2024, a 12-year-old patient fully vaccinated for measles with recent international travel was admitted to an acute care pediatric hospital with fever, rash, mouth sores, and cough and initially thought to have a common viral infection. Rash progression during hospitalization prompted measles polymerase chain reaction (PCR) testing, which was positive. The hospital rapidly conducted contact tracing and infection prevention (IP) efforts, including quarantine, symptom monitoring, and postexposure prophylaxis (PEP) administration, to prevent secondary measles transmission. **Methods:** The patient was placed on airborne isolation ~24 hours after presentation, pending measles testing results. After notification of the positive PCR test, hospital IP staff performed unit walkthroughs and reviewed security footage to retrace the patient’s movements. Staff determined the patient was transported for an echocardiogram, chest radiograph, and walked about the emergency department before isolation. Findings were used to identify contacts requiring quarantine, immunity testing, and PEP. Contacts were notified and those within the PEP window period who were immunocompromised or without presumptive evidence of immunity were offered PEP. All contacts were monitored for development of measles infection. **Results:** Within 36 hours, 158 staff contacts and 90 contacts among patients and visitors were identified, including 9 infants and 24 patients with incomplete measles immunization. At completion, the investigation identified 350 contacts, including 187 staff, 73 patients, and 90 visitors. The hospital administered PEP to 24 staff, 21 patients, and 6 visitors in accordance with American Academy of Pediatrics Redbook recommendations. Among 51 PEP recipients, 2 patients received intramuscular immunoglobulin, 8 patients received intravenous immunoglobulin, and 41 contacts, including all staff and visitors receiving PEP, received MMR vaccine. Six staff members who had no evidence of immunity were furloughed from work for 21 days after index patient contact. No secondary infections were reported. **Conclusions:** A single measles case resulted in 350 contacts among patients, visitors, and hospital staff, exemplifying the broad reach measles can have in healthcare settings. This event highlights the need for a high level of suspicion to promptly identify, isolate, and test possible measles patients. Secondary transmission can be prevented through thorough and coordinated investigations to identify all contacts at the facility, rapidly placing contacts without immunity in quarantine, and mobilizing resources to ensure timely PEP administration to eligible contacts.

